# Downregulation of Candidate Gene Expression and Neuroprotection by Piperine in Streptozotocin-Induced Hyperglycemia and Memory Impairment in Rats

**DOI:** 10.3389/fphar.2020.595471

**Published:** 2021-03-02

**Authors:** Suresh Kumar, Suman Chowdhury, Ajay Razdan, Deepa Kumari, Ram Singh Purty, Heera Ram, Pramod Kumar, Prasunpriya Nayak, Sunil Dutt Shukla

**Affiliations:** ^1^ University School of Biotechnology, GGS Indraprastha University, New Delhi, India; ^2^ Department of Zoology, Jai Narain Vyas University, Jodhpur, India; ^3^ Department of Physiology, All India Institute of Medical Sciences, Jodhpur, India; ^4^ Government Meera Girls College, Mohanlal Sukhadia University, Udaipur, India

**Keywords:** neuroprotection, gene expression, hyperglycemia, piperine, Alzheimer’s

## Abstract

There is accumulating evidence showing that hyperglycemia conditions like diabetes possess a greater risk of impairment to the neuronal system because high glucose levels exacerbate oxidative stress, accumulation of amyloid-beta peptides, and mitochondrial dysfunction, and impair cognitive functions and cause neurodegeneration conditions like Alzheimer’s diseases. Due to the extensive focus on pharmacological intervention to prevent neuronal cells’ impairment induced by hyperglycemia, the underlying molecular mechanism that links between Diabetes and Alzheimer’s is still lacking. Given this, the present study aimed to evaluate the protective effect of piperine on streptozotocin (STZ) induced hyperglycemia and candidate gene expression. In the present study, rats were divided into four groups: control (Vehicle only), diabetic control (STZ only), piperine treated (20 mg/kg day, i.p), and sitagliptin (Positive control) treated. The memory function was assessed by Morris water maze and probe test. After treatment, biochemical parameters such as HOMA index and lipid profile were estimated in the serum, whereas histopathology was evaluated in pancreatic and brain tissue samples. Gene expression studies were done by real-time PCR technique. Present data indicated that piperine caused significant memory improvement as compared to diabetic (STZ) control. The assessment of HOMA indices in serum samples showed that piperine and sitagliptin (positive control, PC) caused significant alterations of insulin resistance, *β* cell function, and insulin sensitivity. Assessment of brain and pancreas histopathology shows significant improvement in tissue architecture in piperine and sitagliptin treated groups compared to diabetic control. The gene expression profile in brain tissue shows significantly reduced BACE1, PSEN1, APAF1, CASPASE3, and CATALASE genes in the piperine and sitagliptin (PC) treated groups compared to Diabetic (STZ) control. The present study demonstrated that piperine not only improves memory in diabetic rats but also reduces the expression of specific AD-related genes that can help design a novel strategy for therapeutic intervention at the molecular level.

## Introduction

There is growing evidence showing a link between Diabetes mellitus (DM) and Alzheimer’s disease (AD). However, the mechanism is not known yet at the molecular level (Butterfield et al., 2014). The intricate link between these two diseases might be due to the dysregulation of blood sugar levels in the body that affects many cells, including brain cells in the central nervous system (CNS) (Malone, 2016). Blood glucose homeostasis is a well-coordinated physiological process controlled by hormone insulin and glucagon released by the pancreas (Roder et al., 2016). Alteration in blood sugar levels in conditions like hyperglycemia deteriorates brain cells and its functions, leading to the development of mild cognitive impairment, which is the early stage of AD (Duarte, 2015). A higher level of glucose not only increases amyloid-beta production in the brain but affects tau phosphorylation in the brain (Mullins et al., 2017). There is accumulating evidence showing that hyperglycemia conditions like diabetes possess a greater risk of impairment to the neuronal system (González-Reyes et al., 2016; Pruzin et al., 2018; Rojas-carranza et al., 2018) because high glucose levels exacerbate oxidative stress, accumulation of amyloid-beta peptides, and mitochondrial dysfunction, and impair cognitive functions, and cause neurodegeneration conditions like Alzheimer’s diseases (Macauley et al., 2015; Gaspar et al., 2016; Kim et al., 2016; Rom et al., 2018; Silzer and Phillips, 2018). It is well established that administration of dexamethasone and streptozotocin (STZ) induced hyperglycemia in rodents (rats), resulting in increased levels of amyloid aggregation, tau phosphorylation, synapses loss, impairment of memory performance, and cognition deficit. STZ is commonly used to induce diabetes in rats. STZ uptake by pancreatic *β* cells is detrimental for *β* cells as STZ causes the generation of reactive oxygen species (ROS) (Vessal et al., 2003). It also results in cognitive impairment, glucose metabolism dysfunction, oxidative stress, and phosphorylation of tau protein resulting in neuronal cell death, a hallmark feature of AD (Salkovic-Petrisic et al., 2013; Moreira-Silva et al., 2018).

Oxidative stress is also responsible for many metabolic and neurologic disorders such as Alzheimer’s disease, Parkinson’s disease, cardiovascular disease, diabetes, and cancer (Kumar et al., 2015). In AD, oxidative stress is triggered by the accumulation of free radicals in the brain that leads to neuronal cell damage. Some of the genes that play a crucial role in amyloid processing, apoptosis, and oxidative stress are BACE1, PSEN1, APAF1, CASPASE3, and CATALASE. Hence, decreasing the expression levels of specific genes involved in apoptosis and oxidative stress pathways by particular therapeutic intervention will be an attractive strategy to counter neurodegeneration in a disease like AD. Several foods, nutrients, and phytocompounds such as resveratrol and curcumin have been investigated for preventive and intervention approaches that might help to prevent diabetes-related cognitive impairment (Mazzanti and Di Giacomo, 2016). Because of these, the present study demonstrated the neuroprotective effects of piperine on a high rich sucrose diet with Dexamethasone and STZ induced hyperglycemia and memory impairment in rats and relevant gene expression profile to address the molecular mechanism underlying the pathway.

## Material and Methods

### Materials

Roswell Park Memorial Institute (culture medium) (RPMI1640), Fetal bovine serum, penicillin (100 U/ml), and streptomycin (100 μg/ml) were purchased from Himedia, India. The general chemicals were also purchased from Sigma or Merck, India. The rat pheochromocytoma (PC12) cells line used in the present study were obtained from the National Center for Cell Science, Pune, India.

### GC-MS Analysis for Authentication of Piperine

The commercially procured piperine sample was identified using GC-MS for its authentication and purity check. GC-MS analysis was achieved using GC-MS-QP2010 Plus, with 230°C and 270°C selected for ion source and an interface temperature, respectively. An Rtx 5 MS capillary column (Restek Company, Bellefonte, United States) with 30 m (length) × 0.25 mm (diameter) × 0.25 μm (film thickness) was used with a solvent cut time of 3.50 min, a threshold of 1,000 eV, and a mass range of 40–650 m/z settings input, as described previously (Chowdhury and Kumar, 2020). In brief, a 260°C injector temperature was programmed from 50°C for 2 min and further increased for up to 250°C with a rate of 4°C/min (3 min hold), followed by an increase of 280°C with a rate of 10°C/min (7 min hold). The peak obtained was compared with the spectrum of the known compounds available in the National Institute of Standards and Technology, U.S. Department of Commerce, and Wiley (John Wiley & Sons Ltd.) libraries.

### Animals and Treatments

#### Development of Type -2 Diabetic Animal Model and Experimental Design

The healthy colony bred albino rats were used to develop a type 2 diabetic animal model with a weight of 150 gm to 200 gm. The rats were fed with a high sucrose diet, and dexamethasone intraperitoneal injections were administered (1.0 mg/kg/day i.e., for 20 days). A single dose of STZ (40 mg/kg) was given on the 21st day to properly develop the diabetic status (Chao et al., 2018). After the development of imbalance in glucose homeostasis through the sucrose feeding with dexamethasone, the mild dose of STZ (40 mg/kg) aggravated the induction of type 2 diabetes and started the treatments of piperine and sitagliptin from the next day as following the modified method of Ghasemi et al. (2014). The diabetic status was confirmed by monitoring glucose levels and insulin production by calculating glucose homeostasis (HOMA). The animals were kept under controlled environmental conditions as per CPCSEA norms. All the experimental protocols were approved by IAEC (Institutional Animal Ethical Committee) registered under the CPCSEA, India (Reg. No.1646/GO/a/12/CPCSEA valid up to 27.03.23).

The experimental design [1s(b)] was categorized into two comparative sets of control groups and treatment groups (Supplementary Material). The control groups consisting of two groups i.e., vehicle control and diabetic control, whereas treatment groups were divided into two groups i.e., piperine treatment groups and sitagliptin treated groups (Positive control). Each group consisted of seven animals (n = 7). The contributing animal groups were categorized as follows.

Group A: Vehicle control (VC)
Group B: Diabetic control (STZ)
Group C: Piperine treatment (STZ/PIP)
Group D: Sitagliptin group (STZ/SITA)

Both piperine (20 mg/kg/day) and Sitagliptin (25 mg/kg/day) were administered daily for 4 weeks during the course of the experiment. The dose of 20 mg/kg/day of piperine is considered the standard dose, also mentioned in the literature (Ren et al., 2018; Guo et al., 2020).

#### Morris Water Maze

To evaluate the neurocognitive damage in these animal groups, a spatial memory task was used. Morris water maze (MWM) was employed to compare the spatial task performances of diabetic rats with that of control animals. The procedure for the training and assessment of behavioral task was carried out as described elsewhere (Dasari et al., 2016).

Animals were allowed to swim in a circular tank filled with water and a hidden (1 cm below the water level) platform in an isolated area. On the first day, animals were dropped on the opposite quadrant (in relation to the hidden platform), and the time required for finding the platform was noted as escape latency. If any animal could not find the platform within 3 min of exposure, they were guided to the platform, and the escape latency was noted as 180 s. Once the animal has reached the platform, it was allowed to rest there for 1 min. On the first day, three training trials were given with a gap of 30 min between tests. However, escape latency (Day 0) was recorded from the first trial only. On the 2nd day, escape latencies (Day 1) of animals were recorded with a single trial releasing the animals from the opposite quadrant. On the third day, escape latencies (Day 2) were noted with releases from the opposite quadrant, left quadrant, and right quadrant with respect to the quadrant where the platform was hidden. The hidden platform was removed on the fourth day (probe test), and the times the animal spent in all the quadrants were noted during a swimming session of 3 min.

##### Serum Biochemistry of Insulin, Glucose and Glucose Homeostasis (HOMA)

The HOMA was calculated for insulin and glucose by following the standard formula of Matthews et al. (1985).

$$\text{HOMA}-\text{IR}=\frac{\text{Fasting Insulin}\left(U/L\right)\times \;\text{Fasting Glucose}\left(\text{mmol}/\text{L}\right)}{22.5}$$

$$\text{Insulinsensitivity}\left(\text{IS}\right)=\frac{1}{\left[(\text{Insulin}\left(\text{U}/\text{L}\right)\times \text{Log}\left(\text{glucose}\left(\text{mmol}/\text{L}\right)\right)\right]}$$

$$\text{HOMA}-\text{β}=\frac{20\;\times \;\text{fasting Insulin}\left(\text{U}/\text{L}\right)}{\text{fasting Glucose}\left(\text{mmol}/\text{L}\right)}-3.5$$

##### Lipid Profile

The lipid profile parameters, total cholesterol, triglyceride, HDL-cholesterol, VLDL-cholesterol, LDL-cholesterol, and atherogenic indices of plasma were calculated by following standard methods using the Fieldward formula (Dobiášová and Frohlich, 2001; Ram et al., 2020).

LDL-C (mg/dL) = TC (mg/dL)-HDL-C (mg/dL)−TG (mg/dL)/5.

##### Histopathology of Pancreases and Brain

The animals were sacrificed after four weeks of experimentation (28 days) by cervical dislocation as per standard norms and further proceeded for histological preparations.

###### Pancreas Histology

The pancreatic histology was evaluated by following the paraffin sectioning following ascending and descending dehydration with hematoxylin-eosin (H&E) staining (Saravanan et al., 2017). The microphotography of stained slides of the pancreatic islets of the Langerhans performed by the Radical microscope (Model No. RXLr-5), India attached with supported software of ProgRes^®^ SpeedXT core 5 by Jenopatric, Germany.

###### Histopathology of Brain Tissues

Paraffin sections were stained with Cresyl violet to study the neurodegenerative and cytoarchitectural changes. 10 µm thick coronal sections were cut using a rotary microtome. Sections passing through the hippocampus (bregma −1.8 mm to −5.8 mm) were stained with cresyl violet.

### Morphological Variables

To measure the effects of various treatments on hippocampal neurons, neuronal cells were subjected to morphological parameters somatic perimeter (μm), somatic area (μm^2^), somatic aspect ratio, somatic compactness, somatic form factor, and somatic roundness. Both area and perimeter can be used to mark the size, and the rest can be assigned to the shape of a neuron. The following table depicts the methods and formulas for calculating the morphological variables (McGarry et al., 2010).

### Cell Counting

Brain areas were identified using the brain atlas, and slides were examined under an Olympus Ch20i microscope. Images were captured, and cell count was performed using ImageJ. Only neurons with clear somatic nuclei and nucleolus were counted. To obtain an unbiased estimate of cell numbers, Abercrombie's correction factor was applied to total cell count, which compensates for the over counting of sectioned profiles, using the equation:

$$\text{P}=\text{A}*\left[\frac{\text{M}}{\text{M}+\text{L}}\right]$$

P is the corrected value, A is the raw density measure, M is the thickness of the section (in micrometers), and L is the average diameter of cell bodies along the axis perpendicular to the section’s plane (Abercrombie, 1946).

### To Determine the Cytoprotective Activity of Piperine Against H_2_O_2_ Induced Oxidative Stress and Aβ_1-42_ Induced Toxicity in PC12 Cells

RPMI-1640 media enriched with 10% heat-inactivated fetal bovine serum and 1% penicillin (100 U/ml), streptomycin (100 U/ml) was used to maintain PC12 cells in a carbon dioxide (CO_2_) incubator at 37°C in a T-25 flask. The protective effect of piperine against H_2_O_2_ induced oxidative stress in PC12 cells was determined by supplementing the cells with different piperine concentrations and performing an MTT assay described previously (Chowdhury and Kumar, 2020). In brief, cells were pre-treated with piperine at concentrations ranging from 1 μM to 0.25 μM for 2 h before exposure to a half-maximal (IC_50_) concentration of H_2_O_2_ (100 μM final concentration). In another experiment, piperine’s protective effect was determined against Aβ_1-42_ (40 μM final concentration) induced toxicity. In this experiment, Aβ_1-42_ pre-formed fibrils were used to induce toxicity to PC12 cells. MTT assay was carried out, and absorption was read at 570 nm using a UV spectrophotometer (Molecular Device Spectramax M3, equipped with Softmax Pro V 5.4.1 software).

### Gene Expression Studies

#### RNA Isolation

Total RNA was extracted from each mice group's cerebral cortex brain tissue using the standard Trizol method (Chomczynski 1993). The RNA quantity and purity were estimated by analyzing their A260/A280 ratio using the NanoDrop spectrophotometer (Thermo scientific). For further confirmation, the isolated RNA was analyzed by 1.2% agarose gel electrophoresis and visualized using the AlphaImager Gel documentation system (Alpha Innotech Corporation, CA, United States).

#### cDNA Preparation

Total RNA isolated from four different treatment groups i.e., untreated group (Vehicle control), Streptozotocin (STZ) treated group (Diabetes control), piperine plus STZ treated group, and sitagliptin plus STZ treated group (Positive control), were used as a template for the cDNA synthesis. Around 4 µg total RNA was used for first-strand cDNA synthesis by following the manufacturer’s protocol.

Quantitative Real-time PCR was performed with the SYBR green PCR master mix kit with the following thermocycling conditions: Gene-specific primers for all the genes were designed manually using the exon-exon junction region amplify, and their specificity was examined through Primer Blast software of NCBI. OligoAnalyzer tool was used to calculate the Tm of the primers. The forward and reverse primers sequences for the following genes were as follows: forward 5′-CTC​TTC​CCA​GGA​CAA​CTC​C-3′ and reverse 5′-TGA​GTG​GCC​TGA​CTT​TTG​AC-3′ for Apaf1, forward 5′-GGA​GCT​TGG​AAC​GCG​AAG​A-3′ and reverse 5′-CAT​CGG​TAC​CAT​TGC​GAG​C-3′ for Caspase3, forward 5′-CAA​GAG​CTG​CTG​TCC​AGG​A-3′ and reverse 5′-GTA​AGG​CAC​AGG​CCG​ATC​A-3′ for Presenilin-1, 5′-ACC​AAC​CTT​CGT​TTG​CCC​AA-3′ and 5′-CAC​CAA​TCA​GTC​CTT​CCG​C-3′ for BACE-1, 5′-CTT​CTG​GAG​TCT​TTG​TCC​AG-3′ and 5′-CCT​GGT​CAG​TCT​TGT​AAT​GG-3′ for Catalase, forward 5′-AGA​TCA​AGA​TCA​TTG​CTC​CTC-3′ and reverse 5′-CGC​AGC​TCA​GTA​ACA​GTC​C-3′ for *β*-actin. *β*-actin was used as an internal control. The melting curve obtained was analyzed, and the relative expression of transcripts was quantified by 2-^ΔΔct^ value method (Livak and Schmittgen 2001).

### Molecular Docking

Docking studies were carried out using the SwissDock server (Grosdidier et al., 2011). The X-ray crystallographic structure of APAF1(PDB code: 1Z6T), BACE1(PDB code: 4D8C), PSEN1(PDB code: 6IDF), Catalase (PDB code: 1TGU) and Caspase 3(PDB code: 3DEK) were retrieved from Protein Data Bank. The missing hydrogen atoms and charges were added by DockPrep application within UCSF Chimera, followed by energy minimization using Gasteiger charges (Pettersen et al., 2004). The structure of piperine was downloaded from PubChem in sdf format and converted to. mol format. Docking of piperine with proteins was carried out using the default parameter of the SwissDock server.

### Statistical Analysis

All the data represent the means ± standard deviation of triplicate determinations. The student t-test was performed for statistical analysis using online software, GraphPad. The significance value of *p* < 0.05 was considered significant, and *p* < 0.01 was considered very significant, and *p* < 0.001 was extremely significant.

## Results

### Characterization of Piperine

**FIGURE 1:**
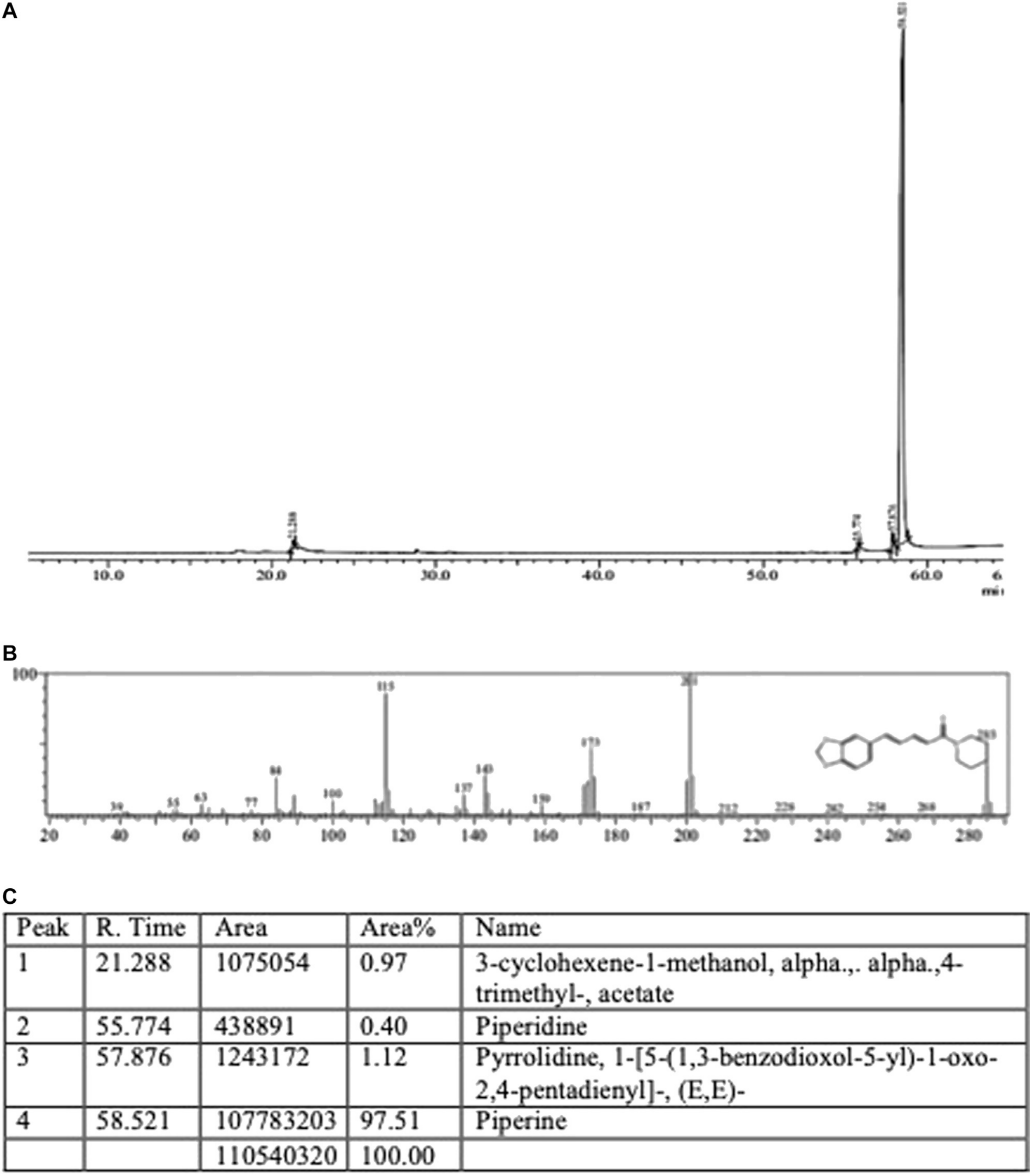
s**(A)** Piperine **(A)** GC-MS chromatogram **(B)** Mass spectra and fragmentation analysis **(C)** Peak report.

For piperine’s characterization, the GC-MS of commercially procured piperine was compared with the fragmentation pattern of piperine structure present in the NSIT library (Figure 1A). The chromatogram shows the presence of three very minor peaks of 3-cyclohexene-1-methanol, alpha, alpha,4-trimethyl-, acetate; piperidine, and pyrrolidine, 1-[5-(1,3-benzodioxol-5-yl)-1-oxo-2,4-pentadienyl]- (E,E) with area percentage of 0.97, 0.40, and 1.12, respectively. Though the sample showed a strong peak of piperine with an area of 97.51%.

### Effect of Piperine on the Viability of PC12 Cell Against H_2_O_2_ Induced Oxidative Stress

**FIGURE 2:**
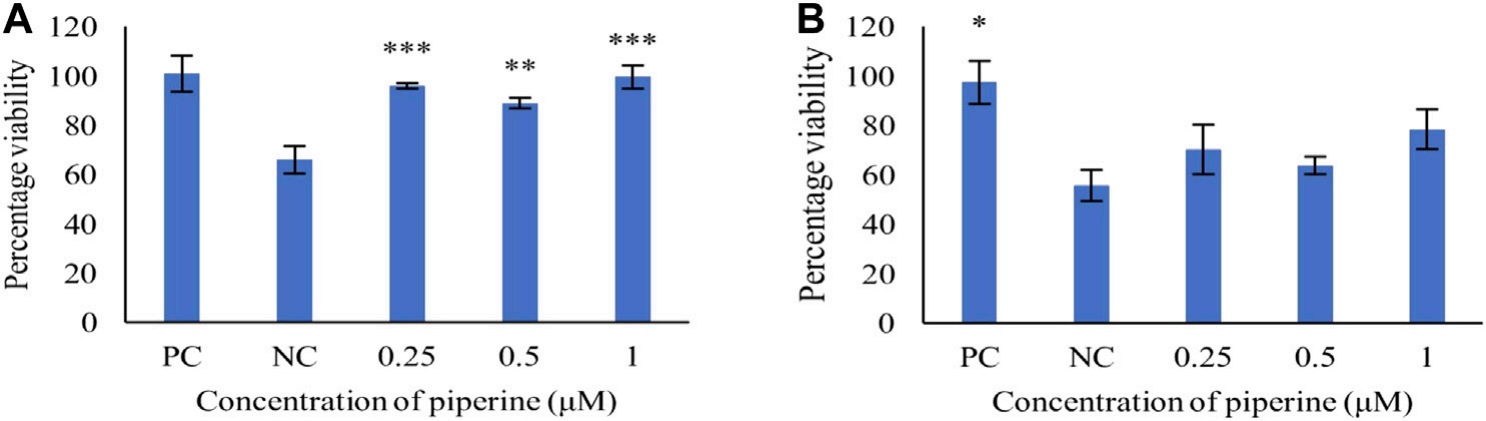
Concentration-dependent protective effect of piperine against **(A)** H_2_O_2_ induced oxidative stress and **(B)** Aβ_1-42_ fibrils induced cytotoxicity in PC12 cells. NC: Negative control (Aβ_1-42_ only), PC: Positive control (No Aβ_1-42_). Data are presented as mean ± SD of three separate experiments performed in triplicate. *p < 0.05, **p < 0.01 with the negative control (NC).

The PC12 cells were pre-treated with piperine, followed by H_2_O_2_ induction to determine the protective effect of piperine against H_2_O_2_ induced oxidative stress. At 1 µM final concentration of piperine, 99.39 ± 4.70% percentage of viability was observed as compared to the negative control (H_2_O_2_ only) (Figure 2A), showing that piperine significantly protected PC12 cells against H_2_O_2_ induced oxidative stress (*p* < 0.01).

### Effect of Piperine on the Viability of PC12 Cells Against Aβ_1-42_ Induced Toxicity

To study the protective effect, PC12 cells were pre-treated with varying concentrations of piperine (0.25–1 μM). The pre-treatment of piperine prior to induction of Aβ_1-42_ fibrils (40 μM), showed concentration-dependent attenuation of Aβ_1-42_ induced toxicity, with maximum cell viability of 78.28 ± 13.90% as compared to the negative control at 1 μM concentration of piperine (Figure 2B).

### Effect on Glucose Homeostasis (HOMA) and Lipid Profile of Serum

#### Effect of Treatments in Glucose Homeostasis (HOMA)

**FIGURE 3:**
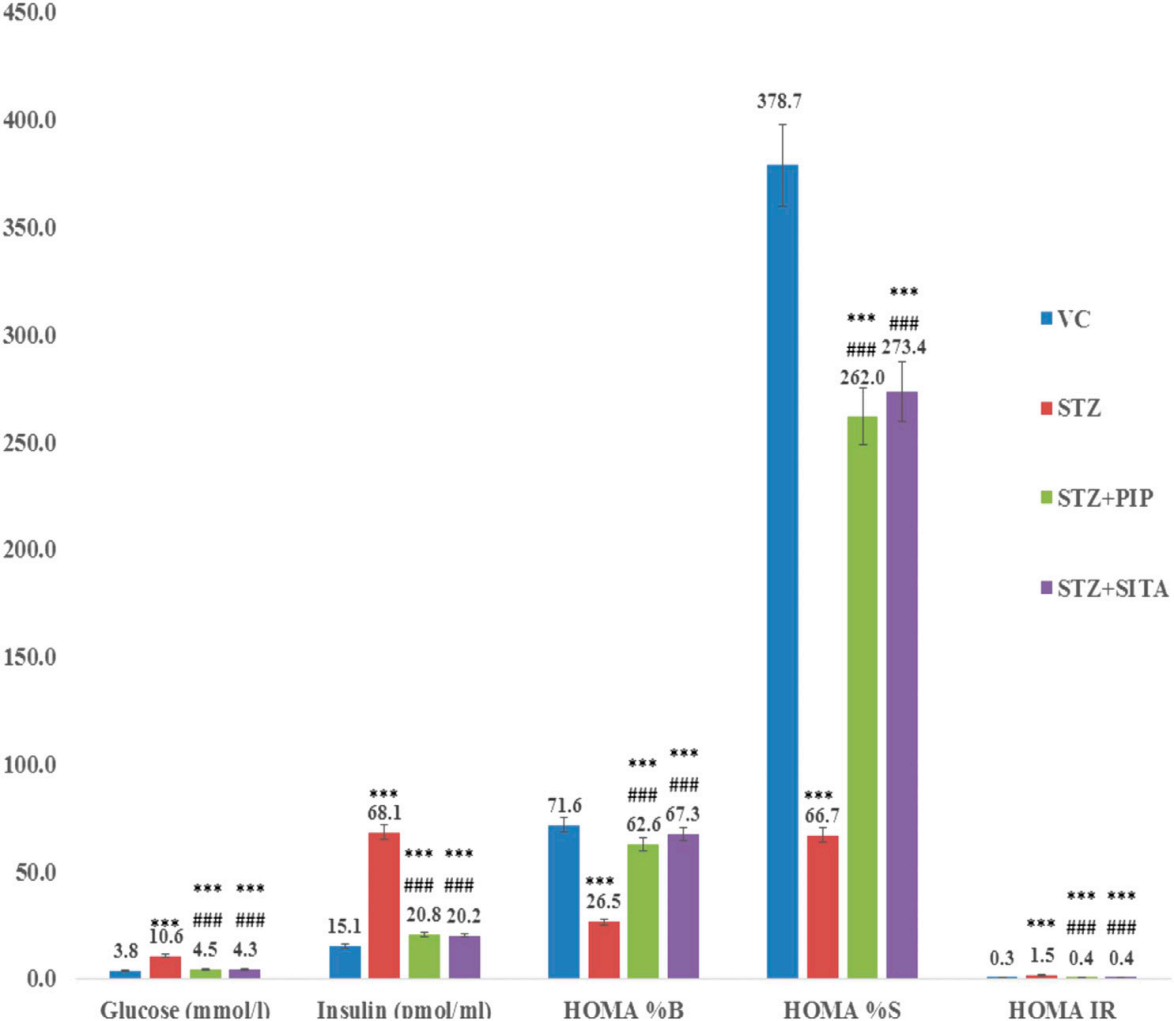
Effect of the piperine on glucose homeostasis (HOMA) (A, B, C, and D were experimental group) (See the experimental design in material and methods section) (Data are means ± S.E.M. (n = 7); *, *p* ≤ 0.05 and c, *** ≤ 0.001 as compared to the respective control values and g, ### ≤ 0.001 and d = nonsignificant as compared to the respective values of the diabetic control group). Blue-Vehicle Control (VC); Red-Diabetic control (STZ); Green-Piperine treatment (STZ + PIP); Purple-Sitagliptin treatment (STZ + SITA).

The significant (*p <* 0.001) increased insulin resistance (IR), *β*-cell function (β%), and insulin sensitivity (S%) were seen in the diabetic group in comparison to the vehicle control group. Accordingly, the insulin and glucose levels were represented its levels in the diabetic group. Although the piperine and sitagliptin, treatments caused significant alterations in HOMA indices of IR, β%, and S%. Accordingly, reductions were observed in levels of insulin and glucose (Figure 3).

#### Effects of Treatments in Lipid Profile and AI

**FIGURE 4:**
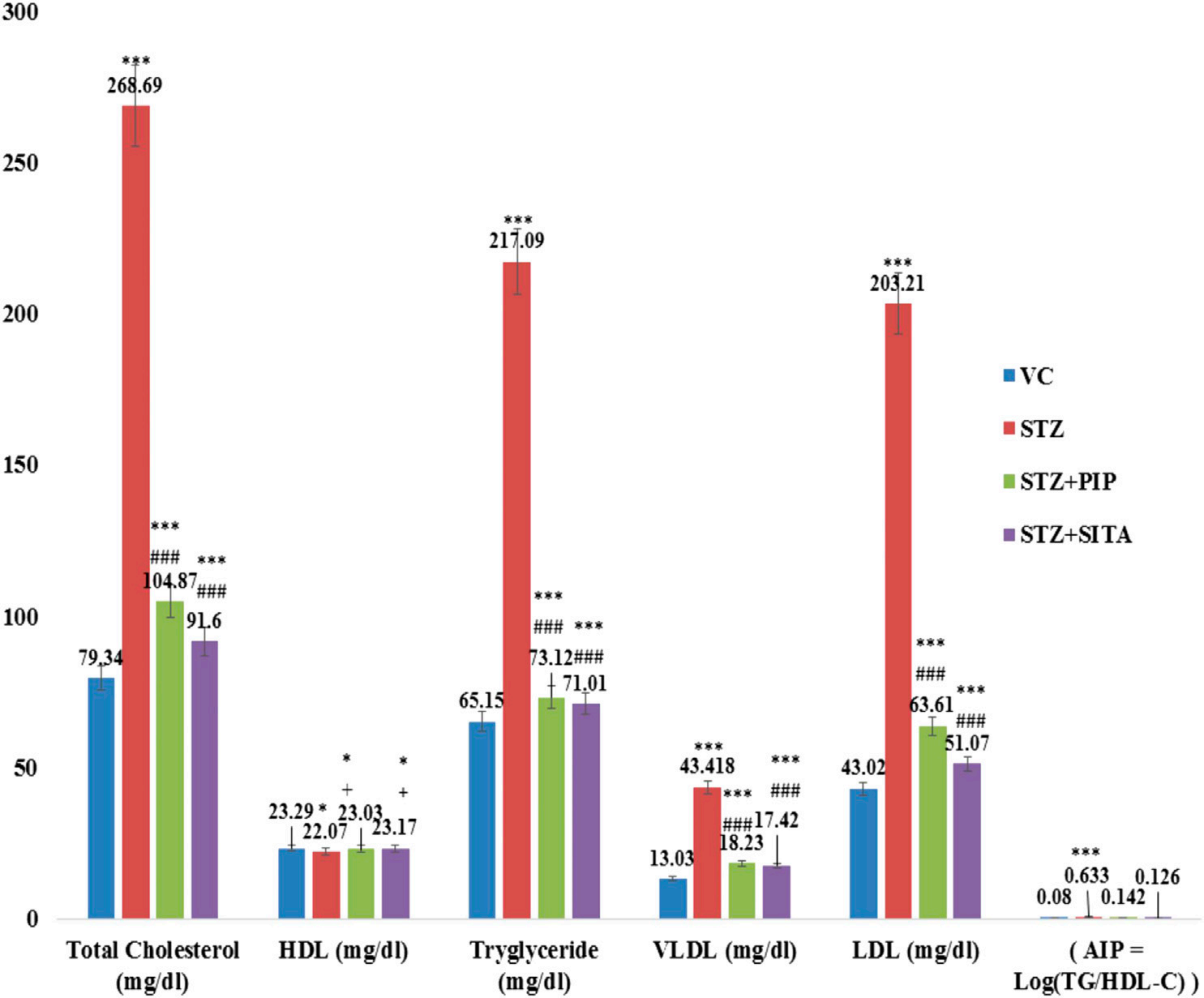
Effect of the piperine on lipid profile and AI (Atherogenic Index) (Data are means ± S.E.M. (n = 7); *, *p* ≤ 0.05 and c, *** ≤ 0.001 as compared to the respective control values and g, ### ≤ 0.001 and d = non-significant as compared to the respective values of the diabetic control group). Blue-Vehicle Control (VC); Red-Diabetic control (STZ); Green-Piperine treatment (STZ + PIP); Purple-Sitagliptin treatment (STZ + SITA).

In the diabetic group, lipid profile (total cholesterol, LDL-cholesterol, VLDL-cholesterol, and triglyceride (Tg)), as well as the atherogenic index (AI = log(Tg/HDL)), were elevated significantly in comparison to the vehicle control group. The piperine and sitagliptin treatments made significant reductions in lipid profile and AI (Figure 4).

### Effects of Piperine on Learning and Memory in STZ-Treated Rats

**FIGURE 5:**
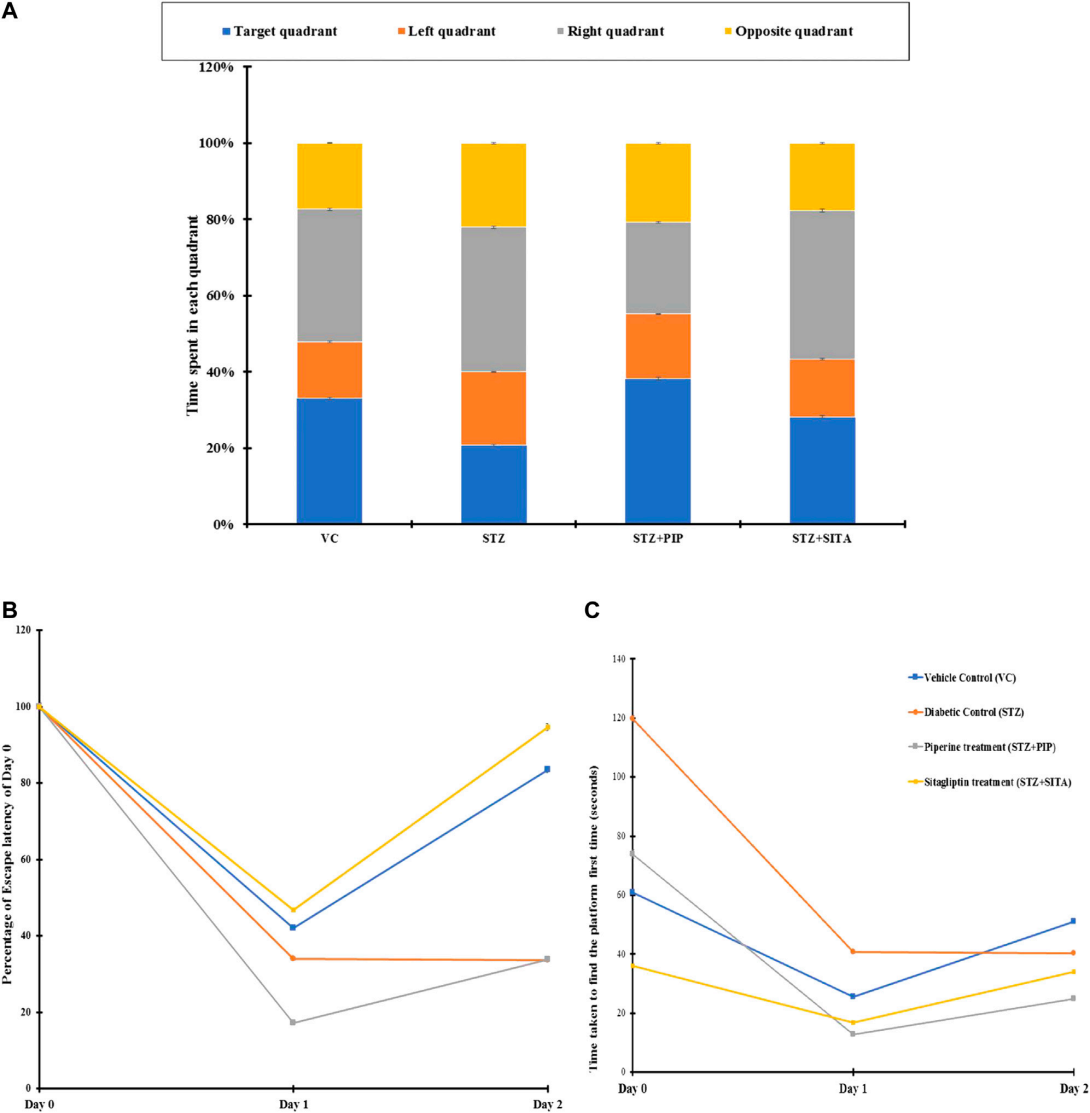
**(A)** Percentage distribution of time spent by the animals in different quadrants in search of the hidden platform (probe test) in MWM. Figure 5B. Day wise changes in time required to find the hidden platform when the animals were released on the opposite quadrant of MWM. Vehicle control (VC); Diabetic control (STZ); Piperine treatment (STZ + PIP); Sitagliptin treatment (STZ + SITA). Figure 5C. Changes in escape latency after 24 h (Day 1) and 48 h (Day 2) in different groups of animals in comparison to their respective first-time exposure (Day 0) to MWM. Vehicle control (VC); Red-Diabetic control (STZ); Green-Piperine treatment (STZ + PIP); Purple-Sitagliptin treatment (STZ + SITA).

The cognitive-behavioral task was evaluated through MWM. The water escape task evaluates spatial learning and memory. On day 1, when the animals were exposed to a circular water tank for the first time, diabetic group B took the maximum time to find the hidden platform. In contrast, the sitagliptin-treated group D took the lowest time, which was even better than the control group of rats A (Figure 5A). The piperine-treated group C demonstrated escape latency that was lesser (38%) than the Diabetic control B but higher (21%) than the group D. Interestingly, the swimming speeds of A, D, and C were in the comparable range (<5% differences), while that of group B was relatively less (∼17%) compared to other groups. Similarly, group B showed the most unsatisfactory performance in terms of path length and cumulative path distance. Contrary to these observations, swim path efficiency was better in group B and group C.

Exposure to the same task after 24 h evaluated the memory function of the animals. The animals of group B took the longest time to escape with the longest path and slowest swimming speed (Figure 5B). Though group B animals’ path efficiencies were very close to that old vehicle control animals, their slow swimming speed increased the nominal high path length (15%) into a considerably higher cumulative distance (100%). On the other hand, PR and SR animals showed very good escape latency, path length, cumulative distance, and swimming path efficiency. Their values were comparable for these parameters, even in the presence of significant differences in swimming speed. The swimming speed in SR was lesser than group C and was comparable to group B.

There were increases in escape latencies, path length, cumulative distance, and decreases in swim path efficiencies for groups A, D, and C on day 3 compared to that2 for the same task performances (Figure 5C). Nevertheless, cumulative distance, path length, and escape latency of group B were high with lower swim path efficiency, in comparison to group D and group C. The speed of swimming in B was in between that of D and C.

When the starting point for the task changed by +90° or −90°, DR animals performed very poorly in swim path efficiency. However, variations in performances of D and C were also noted. Even group A animals also deteriorated in their performances in some cases. A probe test on day 4 was carried out to evaluate the cognitive functions in the absence of the hidden platform. Of all the animal groups, the B group animals spent the minimum time in the target quadrant and maximum time in the opposite quadrant.

### Histopathology

#### Effect of Piperine on Pancreatic Histoarchitecture of the Islet of Langerhans

**FIGURE 6:**
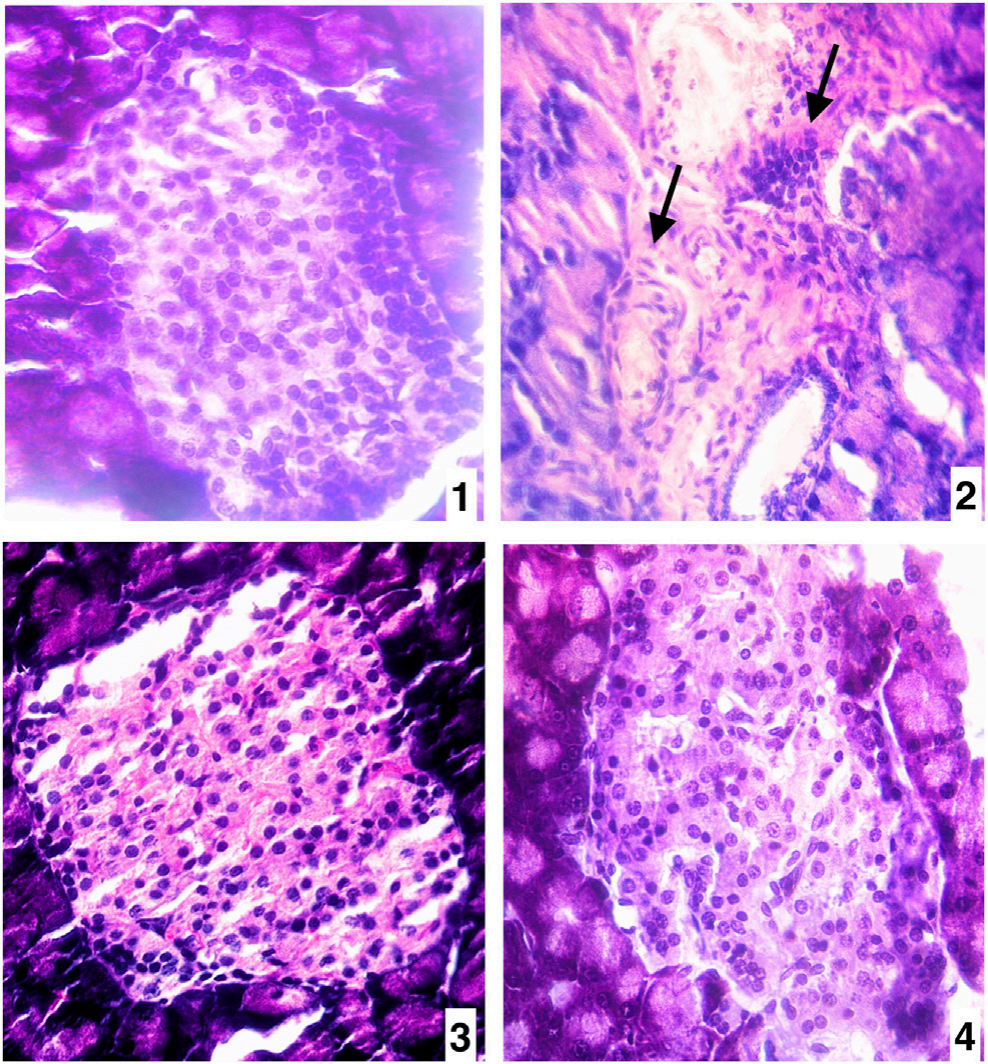
**(A)** Pancreas of vehicle control (VC) (400X H&E) **(B)** Pancreas of diabetic control (STZ)(400X H&E) **(C)** Pancreas of the piperine treatment (STZ + PIP)(400X H&E) **(D)** Pancreas of sitagliptin treatment (STZ + SITA)(400X H&E).

The high sucrose diet and a mild dose of STZ caused significant alterations in histoarchitecture of the pancreas by promoted degenerative changes in the islet of the Langerhans as well as different degrees of necrosis in the nucleus in comparison to the vehicle control group (Figures 6A,B). The degenerative changes also were seen in vascular tissues and connective tissues. Whereas the treatments of piperine and sitagliptin promoted the restoration of histoarchitectures by increasing cellular mass and subsiding the necrosis of nucleus of islet cells with rearrangements of vascular tissues and connective tissues (Figures 6C,D).

#### Effect of Piperine on Brain Tissue Histopathology

Significant differences were observed in CA3 (*Cornu Ammonis* areas) and dentate gyrus (Dg) neurons morphology between the experimental groups. Other regions of hippocampus CA1, CA2, and CA4 have not exhibited any marked changes. The present study shows more significant effects on neuronal soma size as compared to neuronal shape factors.

**FIGURE 7:**
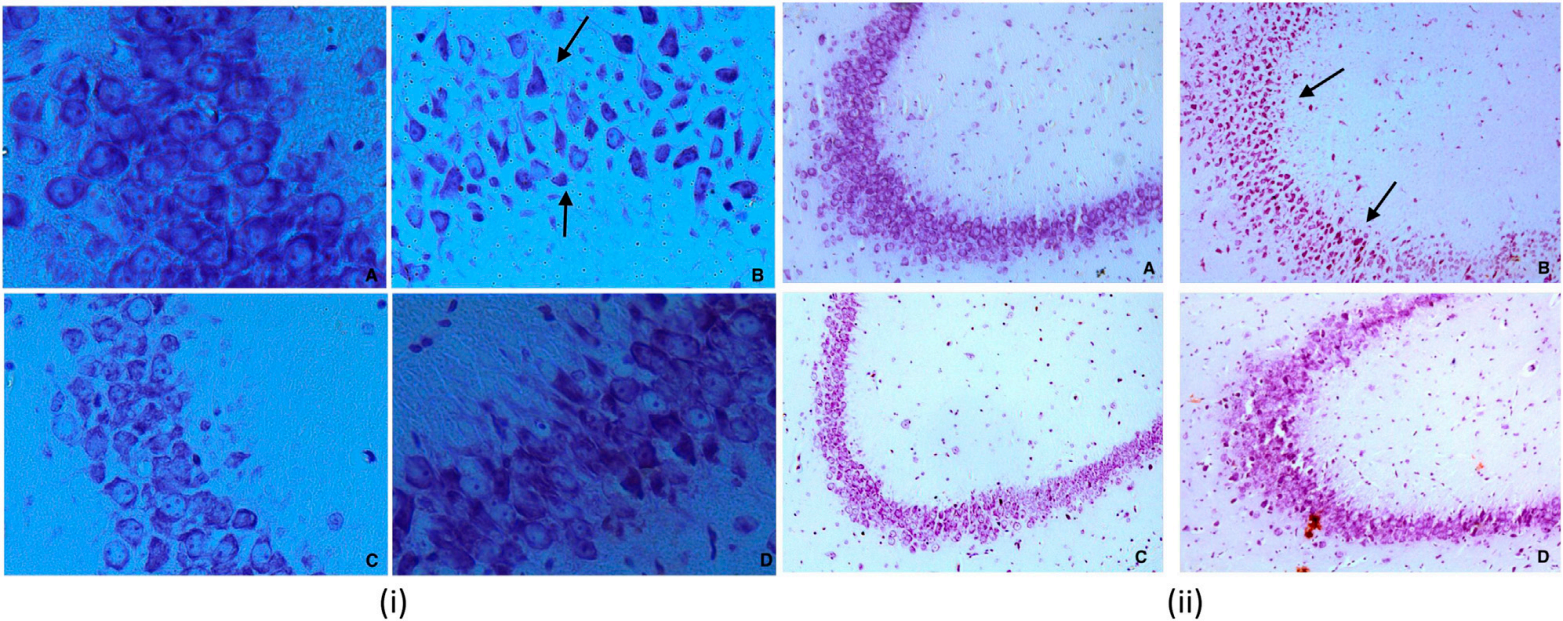
(i) Nissl staining for CA3 sub region of hippocampus for various groups at 40X **(A)** Vehicle control (VC) **(B)** Diabetic control (STZ) **(C)** Piperine treatment (STZ + PIP) **(D)** Sitagliptin group (STZ + SITA). 7 (ii) Nissl staining for CA3 sub region of hippocampus for various groups at 10X **(A)** Vehicle control (VC) **(B)** Diabetic control (STZ) **(C)** Piperine treatment (STZ + PIP) **(D)** Sitagliptin group (STZ + SITA).

Treatment with dexamethasone reduced the size probably because of the degenerative process. In contrast, treatment with piperine and sitagliptin exhibited recovery, and larger soma could be interpreted as protective effects (Figures 7i,ii). Larger soma may have better metabolic and cellular systems required to have better synaptic connections and neuronal activities, which corresponds to enhanced learning ability and memory. SA of CA3 neurons in the Diabetics group was 266.40 ± 10.72 µm^2^, whereas it was 306.73 ± 13.96 µm^2^ for the control group. Treatment with piperine and sitagliptin increased the SA and found to be 279.15 ± 16.71 and 289.15 ± 15.60 µm^2^, respectively. More significant changes were observed in Dg Neurons after dexamethasone treatment SA was decreased by 18.36 percent, and piperine and sitagliptin treatment exhibited SA values matching with control animals (249.15 ± 13.76 and 251.95 ± 12.98), respectively.

**FIGURE 8:**
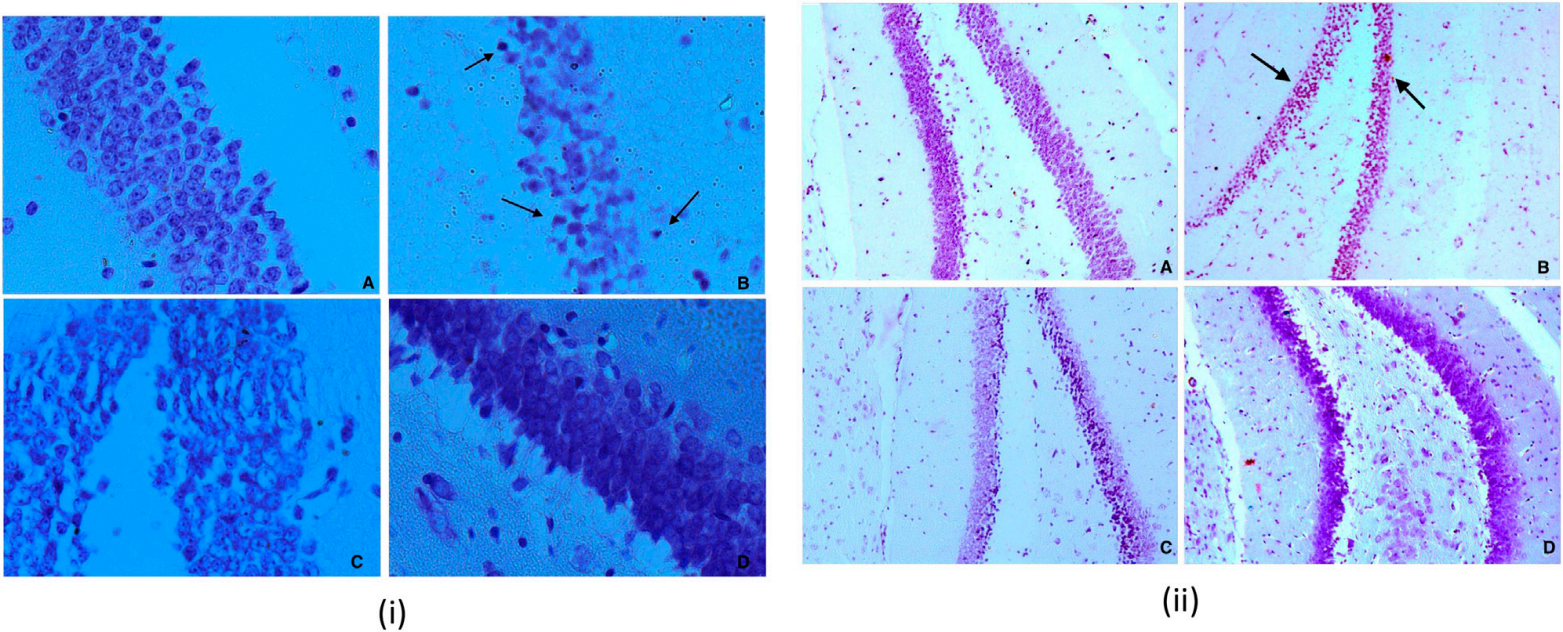
(i) Nissl staining for Dg sub region of hippocampus for various groups at 40X **(A)** Vehicle control (VC) **(B)** Diabetic control (STZ) **(C)** Piperine treatment (STZ + PIP) **(D)** Sitagliptin group (STZ + SITA). 8 (ii) Nissl staining for Dg sub region of hippocampus for various groups at 10X **(A)** Vehicle control (VC) **(B)** Diabetic control (STZ) **(C)** Piperine treatment (STZ + PIP) **(D)** Sitagliptin group (STZ + SITA).

A 23 percent decrease in SP was observed for CA3 neurons in dexamethasone-treated animals. This was found to approximately close to control animals in piperine and sitagliptin treated animals; a change of 10.57 and 3.39 percent was observed, respectively. Similar observations were evident for Dg also SP for control neurons was measured 61.63 ± 3.67 µm and reduced to 51.26 ± 1.16 µm in dexamethasone-treated animals, here no doubt to state that sitagliptin performed better (59.61 ± 3.99 µm) as compared to piperine (56.46 ± 1.35 µm) (Figures 8i,ii].

#### Morphometry

Cell count shows a decrease in number after dexamethasone treatment in CA3 and Dg area; this was further restored with the treatment of piperine and sitagliptin. A significant decrease was observed both in CA3 and Dg neurons after treatment with dexamethasone. A close analysis of data exhibited that both the areas were affected equally with a reduction of 29 and 28 percent, respectively. The total number of neuronal cells in the CA3 and Dg sub-region of the hippocampus of piperine and sitagliptin was approximately closer to control animals, and values for the CA3 sub-region were 22.43 ± 0.40 and 23.03 ± 0.55 (Table 1). For the Dg sub-region, the total number of cells in piperine and sitagliptin treated animals was 38.76 ± 1.05 and 38.96 ± 1.19, respectively (Table 2). The decrease in cell number indicates cell death, and protective effects are indicated by the restored number of cells compared to control animals.

**Table:** 

S. No	Morphological variable name	Abbreviation	Formula
1	Somatic perimeter (μm)	SP	The perimeter of the Soma
2	Somatic area (μm^2^)	SA	Area of Soma
3	Somatic aspect ratio	SAR	Max diameter of soma/min diameter of soma
4	Somatic compactness	SCom	[(4/π)*Area)^1/2^]/max diameter
5	Somatic form factor	SFF	(4π*Area)/(Perimeter^2^)
6	Somatic roundness	SRo	(4*Area)/(π* max diameter^2^)

**TABLE 1:** Measurements of the morphological size and shape of CA_3_ neurons.

CA3	Control	Diabetic	Piperine	Sitagliptin
Area	306.73 ± 13.96	266.40 ± 10.72*	279.15 ± 16.71	289.15 ± 15.60
Perimeter	115.44 ± 7.90	95.13 ± 7.58	103.23 ± 9.73	111.52 ± 7.20
Cell count	24.99 ± 0.21	17.66 ± 0.26**	22.43 ± 0.40**	23.03 ± 0.55**
Somatic aspect ratio	1.27 ± 0.042	1.40 ± 0.05	1.32 ± 0.04	1.29 ± 0.05
Somatic compactness	1.02 ± 0.03	1.09 ± 0.04	1.01 ± 0.06	1.00 ± 0.04
Somatic form factor	0.47 ± 0.08	0.79 ± 0.3	1.60 ± 0.98	0.48 ± 0.09
Area	1.08 ± 0.07	1.25 ± 0.10	1.14 ± 0.17	1.06 ± 0.09

Significance level *p value < 0.05, **p value < 0.01.

The morphological shape of cell bodies was analyzed based on the somatic aspect ratio (SAR), somatic circularity index (SCI), and somatic roundness (SRo). SAR exhibits the symmetry of cellular shape. SAR value close to 1 is indicative of circular or spherical shape. CA3 neurons exhibited higher SAR values, and this was further increased in dexamethasone-treated animals (1.40 ± 0.05), indicative of a disruption of pyramidal shape. Animals treated with piperine and sitagliptin exhibited SAR values close to control animals, reflecting the pyramidal shape’s retention. As expected, Dg neurons exhibited SAR values close to 1.0, and no significant difference was observed between various experimental groups. We found no significant changes in the SCom, SFF, SRo, suggesting that the soma size changes were uniform rather than shrinkage or expansion along a particular axis.

### Gene Expression Analysis in the Cerebral Cortex Brain Tissue of Rat

**FIGURE 9:**
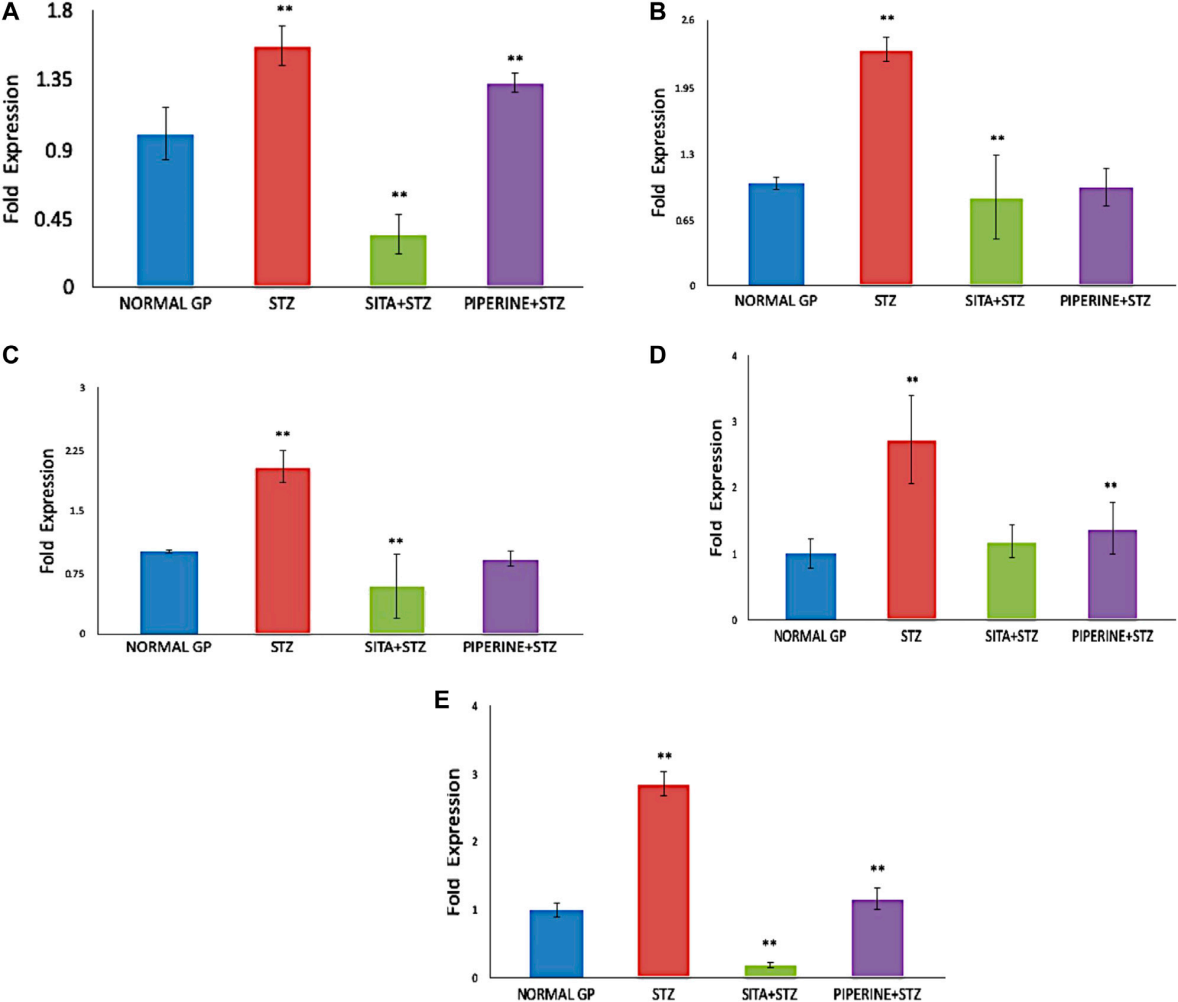
Gene Expression analysis in the cerebral cortex brain tissue of rat (NORMAL GP = Control group; STZ = Streptozotocin treated group; SITA + STZ = Streptozotocin and sitagliptin treated group; PIPERINE + STZ = Streptozotocin and piperine treated group). In compared to the untreated control group, the expression of *BACE1*, *PSEN1*, *APAF1*, *CASPASE3*, and *CATALASE* genes were significantly upregulated (*p* ≤ 0.05) in STZ treated group (Diabetes control). However, when the STZ treated group (Diabetes control) was treated with either sitagliptin (Positive control) or piperine, the expression of all the five genes were significantly down-regulated (*p* ≤ 0.05). Data are presented as mean ± SD. **p < 0.01 with the control (NORMAL GP).

For gene expression analysis, total RNA was isolated from the cerebral cortex, and its quality was analyzed by assessing the RNA purity and integrity. The absorbance ratio A260/280 ratios were greater than 1.9 of all the RNA samples. The sharp bands for 28S and 18S ribosomal RNA were observed when separated in 1.2% agarose gel electrophoresis, indicating the good quality RNA. Using the manufacturer instructions, cDNA was prepared and used as a template for gene expression analysis. Using the Real quantitative Time-PCR, the expression of selected genes, including *BACE1, PSEN1, APAF1, CASPASE3, and CATALASE*, in rat's cerebral cortex brain tissue was analyzed in four different treatment groups. In STZ treated group (Diabetes control), the expression of *BACE1, PSEN1, APAF1, CASPASE3, and CATALASE* genes were upregulated in the cerebral cortex of the rat brain as compared to the untreated group (Vehicle control) (Table 3). The fold change increase in these genes’ expression levels varied, and it ranged between 1.5 and 2.8-fold increases (Figure 9). However, their expression decreased drastically when treated with sitagliptin (Positive control). Reduction percentage in the expression of these genes ranged between 59 and 96% decreases in sitagliptin treated (Positive group) in comparison to the STZ treated group (Diabetes control). Upon treatment with piperine, the expressions of these genes were significantly decreased (*p* ≤ 0.05). When these genes’ expression was compared between the piperine treated group and the Diabetes control group, statistically significant changes (*p* ≤ 0.05) were observed.

**TABLE 2:** Measurements of the morphological size and shape of Dg neurons.

Dg	Control	Diabetic	Piperine	Sitagliptin
Area	272.94 ± 12.06	222.75 ± 9.71**	249.15 ± 13.76	251.95 ± 12.98
Perimeter	61.63 ± 3.67	51.26 ± 1.16**	56.46 ± 1.35	59.61 ± 3.99
Cell count	43.33 ± 0.80	31.00 ± 0.67**	38.76 ± 1.05**	38.96 ± 1.19**
Somatic aspect ratio	1.03 ± 0.03	1.01 ± 0.05	1.01 ± 0.03	1.02 ± 0.02
Somatic compactness	1.08 ± 0.02	1.20 ± 0.09	1.24 ± 0.05*	1.15 ± 0.03
Somatic form factor	1.13 ± 0.14	1.11 ± 0.07	1.03 ± 0.07	2.78 ± 1.56
Somatic roundness	1.20 ± 0.06	1.68 ± 0.30	1.64 ± 0.15**	1.36 ± 0.08

Significance level *p value < 0.05, **p value < 0.01.

### Molecular Docking Analysis

**FIGURE 10:**
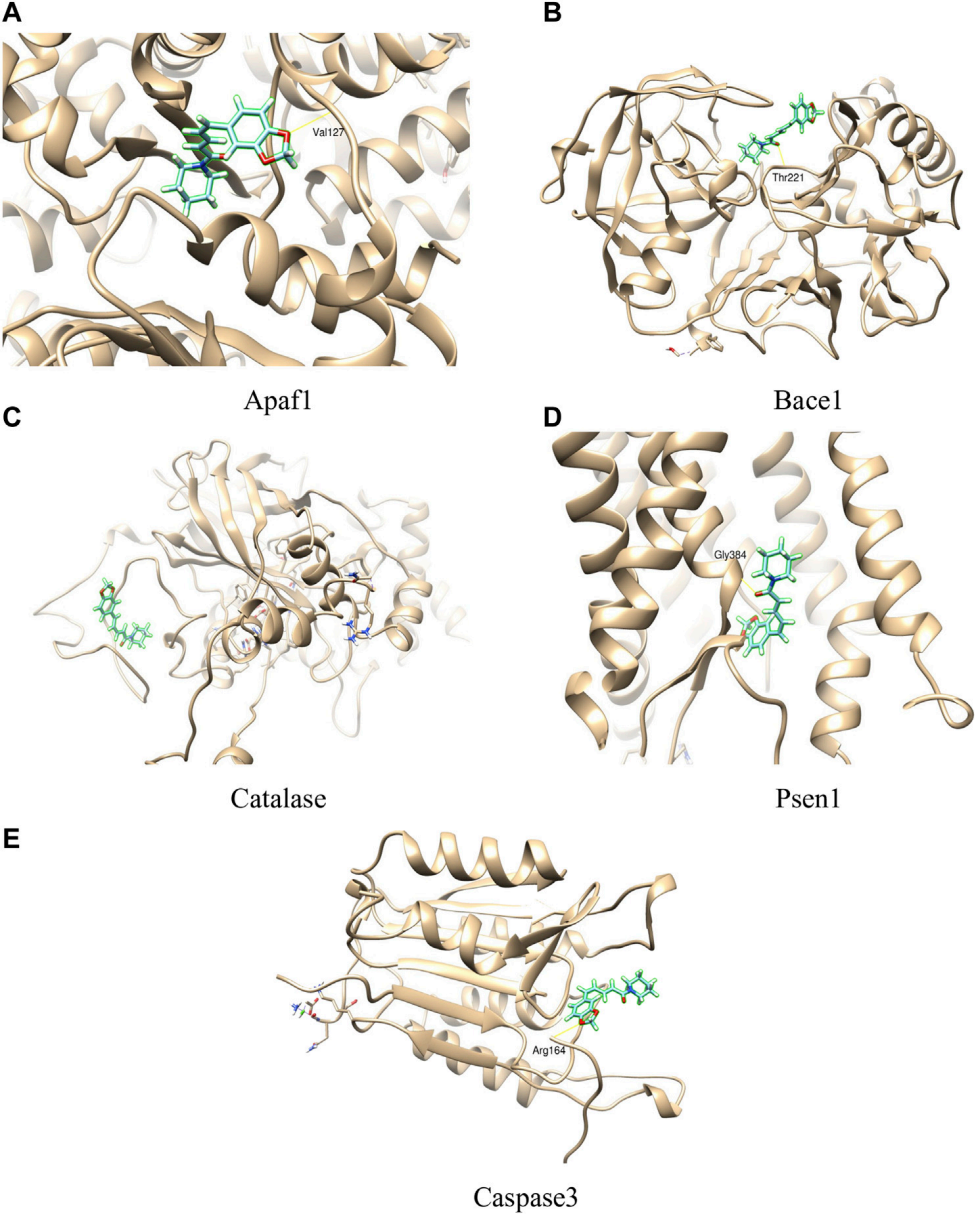
Molecular interactions of piperine with different proteins (A = APAF1, B= BACE1, C= CATALASE, D = PSEN1, E = CASPASE3).

In the present study, the interaction between piperine and some proteins involved in amyloid processing, apoptosis, and oxidative stress such as BACE1, PSEN1, APAF1, CASPASE3, and CATALASE were studied to explore the binding mode, using SwissDock server. The binding affinity of the proteins with piperine was measured by FullFitness score and deltaG (kcal/mol). The FullFitness score was highest for APAF1 with −8.02 kcal/mol deltaG followed by CATALASE (−7.51 kcal/mol), PSEN1 (−7.35 kcal/mol), BACE1 (−7.20 kcal/mol), and the least score was found CASPASE3 (−6.55 kcal/mol) as shown in Table 4 and Figure 10. Besides, hydrogen bond formation was seen in all four proteins except CATALASE when docked with piperine. The molecular docking study predicted a binding interaction between each protein and piperine ligand, validated by hydrogen bonding between the proteins and piperine. Molecular docking of piperine with APAF1 revealed H-boding with Val127 of α/β fold of protein (Figure 10A). Docking analysis of BACE1 revealed that Thr221 residue participates in forming H-bond with piperine. Thr221 was also found to interact with the ligand of the X-ray crystal structure of BACE1 (PDB code: 4D8C); therefore, this interaction plays an important role (Figure 10B). In CATALASE protein, no single H-bond interaction was found to be formed with the catalytic residues (Figure 10C). The binding interaction of PSEN1(PDB code: 6IDF) revealed that Gly384 residues are involved in H-bond formation with piperine (Figure 10D). CASPASE 3 was found to interact with amino residue Arg 164, to form H-bond interaction with piperine CA Figure 10 (Molecular docking of piperine with APAF1 revealed H-boding with Val127 of α/β fold of protein (Figure 10A). Docking analysis of BACE1 revealed that Thr221 residue participates in forming H-bond with piperine. Thr221 was also found to interact with the ligand of the X-ray crystal structure of BACE1 (PDB code: 4D8C); therefore, this interaction plays an important role (Figure 10B). In CATALASE protein, no single H-bond interaction was found to be formed with the catalytic residues (Figure 10C). The binding interaction of PSEN1(PDB code: 6IDF) revealed that Gly384 residues are involved in H-bond formation with piperine (Figure 10D). CASPASE 3 was found to interact with two amino acid residues, Glu 396 and Thr 467, to form an H-bond interaction with CA (Figure 10E).

**TABLE 3:** Gene expression profile.

Groups	Genes				
	Caspase 2	PSEN1	BACE1	Apaf 1	Catalase
Control (NORMAL GP)	↓	↓	↓	↓	↓
Diabetic control (STZ)	↑	↑	↑	↑	↑
Positive control (SITA/STZ)	↓	↓	↓	↓	↓
Piperine (PIPERINE/STZ)	↓	↓	↓	↓	↓

**TABLE 4:** Molecular docking result of piperine in terms of full fitness and estimated ΔG values predicted by SwissDock.

Protein	deltaG (Kcal/mol)	FullFitness	Interacting residues	Bond length (Å)
Apaf1	−8.02	−3452.89	Val 127	2.275
Catalase	−7.51	−2139.90	—	—
Psen1	−7.35	−1129.81	Gly384	2.049
Bace1	−7.20	−1529.14	Thr 221	2.216
Casapase3	−6.55	−1380.32	Arg164	2.685

## Discussion

AD and DM are two independent metabolic syndromes but recently reported increasing evidence shows a link between AD and DM (Akter et al., 2011). The complications associated with DM leads to a hyperglycemic condition. The increase in blood sugar level affects neuronal cells. The higher blood sugar levels lead to faster cognitive decline and neuronal cell death. The increased blood glucose levels also result in higher glycation end-products (AGEs) that damage the neuronal cells by inducing oxidative stress, promoting inflammation, and causing direct neurotoxicity to brain cells. The other pathway involved in neuronal cell death is apoptosis (Singh et al., 2014). The administration of dexamethasone and STZ increases oxidative stress in the rat brain. This oxidative stress causes ROS accumulation in the brain that further damage cellular components such as proteins and neuronal cells’ membranes (Uttara et al., 2009). STZ treatment produces free radical’s generation causing oxidative stress, mitochondrial abnormalities which further trigger caspase mediated apoptotic cell death, neuroinflammation which are collectively detrimental to neuronal function which further implicate in the AD pathology (Rai et al., 2013). STZ produces brain insulin resistance or impaired insulin receptor in the brain which leads to Aβ accumulation forming senile plaques and increased tau-phosphorylation forming neurofibrillary tangles by increasing the activity of GSK-3β that results in neuronal and synaptic dysfunction, memory impairment which further leads to AD like pathology (Shingo et al., 2012; Stöhr et al., 2013). STZ exacerbates the pathological changes associated with AD such as amyloidogenic processing of APP, glucose metabolism, insulin signaling, synaptic function (Chen et al., 2014; Zhang et al., 2015).

This study evaluated the neuroprotective effects of piperine and expression of five candidate genes (*BACE1, PSEN1, APAF1, CASPASE3, and CATALASE*) in rat’s cerebral cortex induced hyperglycemia with STZ rat model. Our results showed that all five genes *BACE1, PSEN1, APAF1, CASPASE3, and CATALASE* were significantly down-regulated in the piperine treated group compared to the Diabetic rats (STZ induced) group. It has been reported that piperine has the ability to enhance the bioavailability of compounds by several different mechanisms such as modulation of cell signal transduction, DNA receptor binding, inhibition of drug efflux pump and increasing the absorption of drugs (Bajad et al., 2001). Moreover, study suggested that piperine strongly inhibits human P-glycoprotein and cytochrome P4503A4 (Bhardwaj et al., 2002). A previous study reported the protective role of Piperine in cerebral ischemia induced inflammation rat model by downregulating the expression of Cox-2, NOS-2 and NFKB (Vaibhav et al., 2012). The neuroprotective effect of Piperine in 6-OHDA induced Parkinson’s rat model by its anti-apoptotic and anti-inflammatory activity (Shrivastava et al., 2013). Piperine attenuated Trauma brain injury (TBI) by downregulating the expression of TNF-α, IL-1β and BDNF in TBI mice (Song et al., 2020). A more recent study showed that Piperine treatment enhances memory performance and improves myelin repair in LPC induced demyelination rat model. (Roshanbakhsh et al., 2020). The present study substantiates the claim that piperine can binds to the molecular targets as mentioned in the previous studies mentioned above and can have effect of CNS related neuroprotective effects.

AD is characterized by neuritic plaques, the main pathological hallmarks of AD, primarily consist of amyloid *β* (Aβ) peptides. Aβ peptides are generated by the non-amyloidogenic pathway when amyloid precursor protein (APP) was cleaved by an enzyme called *β*-site APP cleaving enzyme 1 (BACE1). BACE1 plays an important role in APP processing (Resende et al., 2008; Da Costa Dias et al., 2011). Expression of the *β*-secretase gene is implicated in late-onset Alzheimer’s Disease (LOAD) because of its function in initiating Aβ production of PSEN1 (Presenilin 1) component of γ secretase enzyme. *BACE1* gene expression is upregulated in oxidative stress, ischemia, and hypoxia (Guglielmotto et al., 2009). BACE1 cleavage is also affected by a mutation in gene PSEN1 and PSEN2, resulting in the overproduction of Aβ isoforms. Aβ induced toxicity ultimately leads to neuron cell death by activating caspases 3, 6, and 7 (Elmore, 2007). Piperine downregulated both BACE1 and PSEN1 gene expression as compared to STZ treated group. Another, pro-apoptotic genes such as *APAF1* and *CASPASE* are involved in the apoptotic pathway. Gene expression study showed increased expression of pro-apoptotic genes such as *CASPASE* and *APAF1* involved in neuronal cell death via an apoptotic pathway in the AD brain (Papaliagkas et al., 2007). *APAF1* is the gene responsible for encoding a multiprotein complex that plays a crucial role in cell death's mitochondrial pathway. The role of *APAF1* is not very clear in neurodegeneration, although evidence has shown a correlation between *APAF1* and *CASPASE*. The activated Apaf1 is required for the downstream executioner caspase family of genes (Shakeri et al., 2017). Caspase-3 is a cysteine protease family of genes. Many caspases are involved in initiating the signaling and execution of the apoptosis pathway. Caspase-3 is an executioner caspase an important component of the apoptosis pathway (Shi, 2004). It has been found that the activation of caspase-3 is an early event in the pathogenesis of AD. Activation of caspase-3 results in morphological changes leading to neuronal cell death. *In vitro* and *in-vivo* studies suggested that Caspase-3 has a significant role in the neuronal cell death associated with a high level of expression and activation of caspase-3 seen in AD models (Gorman et al., 1998). Gene expression study showed increased expression of pro-apoptotic genes such as caspases and apaf1 are involved in neuronal cell death via an apoptotic pathway in the AD brain (Fortin et al., 2001; Papaliagkas et al., 2007). Both Apaf1 and caspase gene expression were significantly downregulated in the piperine treated group compared to the diabetic control group (STZ treated). The biological system has an antioxidant defense system consists of an enzyme such as catalase widely distributed in the human body (Chen et al., 2012). Catalase breaks down two hydrogen peroxide into oxygen and water using iron or manganese as a cofactor and thus protects cells (Nandi et al., 2019). The catalase gene expression was significantly increased in the group treated with STZ alone (Diabetic control), whereas it was significantly down-regulated in the piperine treated group.

*In vivo* experiments in the diabetic rat model demonstrated improvement of memory functions in the piperine treatment group was observed in neurocognitive function assays. The piperine group showed a relatively shorter time to find the hidden platform in the Morris Water Maze test. However, the escape latency for this group was not better than the diabetic group of animals. Similarly, the memory function of the piperine treated group of animals was also found to be improved compared to that of the diabetic group. This has been further confirmed by the probe test, where similar effects of piperine treatment were reflected.

The increased glucose and insulin levels with abnormal HOMA indices seen in a diabetic animal model may be following the decreased uptake of glucose and insulin resistance, which is further seen in degenerative cellular changes in pancreatic tissues. Several studies reported that increased insulin resistance caused reductions in glucose uptake, which also resulted in apoptosis in several tissues, as seen in pancreatic tissues of the diabetic animal model (Borona, 2008; Khan et al., 2016). The treatments of piperine and sitagliptin caused significant alterations in HOMA indices of IR, β%, and S%, which indicate the interferences of piperine in the metabolism of glucose as well as insulin action. Accordingly, it is a well-established illustration that dietary or orally administered supplement stimulates insulin secretion by increasing GLP-1 activity through gut action (Girard, 2008; Mudaliar and Henry, 2012; Brandt et al., 2018). Supportively, the glucose transporter isoform GLUT4 might be a critical target to treat insulin resistance. Meanwhile, GLUT4 is an insulin-dependent isoform that is accountable for furthermost insulin-encouraged glucose uptake (Abdel Aziz et al., 2020). Consequently, the treatments caused significant reductions in lipid profile and the atherogenic index, which may follow the subside of the gluconeogenesis and inhibition of lipid biosynthesis (Bano, 2013; Petersen and Shulman, 2018). Accordingly, the pancreatic tissues gained restorations in histoarchitecture, which indicates the interference of stimulation may produce by the piperine to increase islet cell mass, which is also seen in reduced insulin resistance and related HOMA indices. It is reported that postprandial dietary stimuli promote the secretion of GLP-1 and GIP by gut cells, which further stimulate insulin secretion and restoration or regeneration of islet *β*-cells (Wu et al., 2015; Prassannaraja et al., 2020).

There were significant differences in hippocampal CA3 and Dg neuronal morphology between the experimental groups (Tables 1, 2; Figures 7, 8). Here we have used a chronic dose of dexamethasone and a single mild streptozotocin dose to induce Hyperglycemia and Insulin resistance. Diabetics’ control animals exhibited changes both in neuronal morphology and number. Histology revealed cell death both in CA3 and Dg sub-regions of the hippocampus. Dexamethasone treatment is comparable to adrenalectomy and leads to corticosteroid depletion and cell death in the hippocampus’s Dg (Hassan et al., 1996; Hornsby et al., 1996), a phenomenon that is well documented after adrenalectomy (Sloviter et al., 1993). Streptozotocin can also induce cell death in the hippocampus, especially in CA3 neurons, by altering the brain antioxidant status and energy impairment (Salkovic-Petrisic et al., 2013). Neuronal damage in the hippocampus contributes to cognitive brain dysfunctions (Agrawal et al., 2009; Saxena et al., 2010).

Nazem et al. (2015) suggested that neuroinflammation leads to the accumulation of Aβ and tau and ultimately leading to Alzheimer’s. Streptozotocin treated animals exhibit an insulin-resistant brain state, which promotes the accumulation of tau and Aβ (Salkovic-Petrisic et al., 2013). Our *in vivo* results also confirm this streptozotocin up-regulated *BACE1, PSEN1, APAF1, CASPASE3, and CATALASE genes* in the animal model. Molecular interaction between piperine and protein of these genes was also studied using molecular docking. Antioxidants are considered to be good neuroprotectants and can ameliorate cognitive impairments. Quercetin, a flavonoid, is useful in the amelioration of STZ-induced cognitive impairment (Liu et al., 2012). Piperine also possesses antioxidant properties and can cross the blood-brain barrier (Srinivasan, 2007; Elnaggar et al., 2015). The beneficial effects of piperine on neurons’ morphology and cognitive functions can be attributed to inherent antioxidant capabilities. The down-regulation of *BACE1, PSEN1, APAF1, CASPASE3, and CATALASE genes* in the animal model and binding of piperine with protein in the *silico* model, shows that the neuroprotective activity of piperine may be closely related.

In cells, Aβ_1-42_ toxicity causes deterioration of chaperoning and proteasomal processing involved in clearance of deposited peptides (Mena et al., 2009). To overcome this failure, cytoprotective property elucidation of a molecule is necessary. This cytoprotective effect of piperine was demonstrated in PC12 cells, which is the standard *in vitro* model used to study neuroprotective compounds. Piperine showed significant protection of PC12 cells against both H_2_O_2_ and Aβ_1-42_ induced toxicity under *in vitro* conditions. Understanding molecular interaction with protein targets and genes regulated by the piperine can provide information regarding its mechanisms of action. Additionally, drug targets do not always affect gene expression changes; they work via signaling cascades. Hence, drugs act on their drug targets and modulate the signaling mechanism (Xie et al., 2016; Makhouri and Ghasemi, 2018). Therefore, we performed *in silico* molecular docking of piperine with a protein involved in amyloid processing, apoptosis, and oxidative stress, and suggested interaction of piperine with important amino acid residues. In the present study, *in silico* studies used was molecular docking approach. Molecular docking is powerful computation tool to understand the structural and chemical basis of ligand-target specificities. The molecular docking results showed the interaction between piperine and specific proteins involved in amyloid processing, apoptosis, and oxidative stress such as *BACE1, PSEN1, APAF1, CASPASE3, and CATALASE*. The binding affinity of the proteins with piperine was measured by FullFitness score and deltaG (kcal/mol). As these proteins are encoded by specific genes, which express these proteins BACE1, PSEN1, APAF1, CASPASE3, and CATALASE, the gene specific primers were designed and gene expression studies using RT-PCR showed alteration of the gene expression profile in the piperine treated group of the abovementioned genes as compared to control. As the genes control most of the biological function through specific proteins, the molecule which can either bind to protein or alter gene expression can be used for therapeutic intervention in amelioration complex conditions line hyperglycemia induced neurodegeneration process. The gene expression profile in response to the piperine support the docking results and propose a credible viewpoint on the pathways associated with protein responses to piperine binding in AD-related drug targets.

## Conclusion

In conclusion, piperine demonstrated to possess neuroprotective properties in STZ induced diabetic rodent model as it can be used as an interventional strategy to protect neuronal cells in hyperglycemic conditions. The improvement of HOMA indices, histo-architectures, and downregulation to genes involved in apoptosis and oxidative stress are scientific evidence underlying the protective mechanism.

## Data Availability

The authors confirm that the data supporting the findings of this study are available within the article and its upplementary aterials.
