# Supplementary figures and images for: Downregulation of Candidate Gene Expression and Neuroprotection by Piperine in Streptozotocin-Induced Hyperglycemia and Memory Impairment in Rats
Source: Front Pharmacol. 2021 Mar 2;11:595471. doi: 10.3389/fphar.2020.595471 (PMC7962412; doi:10.3389/fphar.2020.595471)

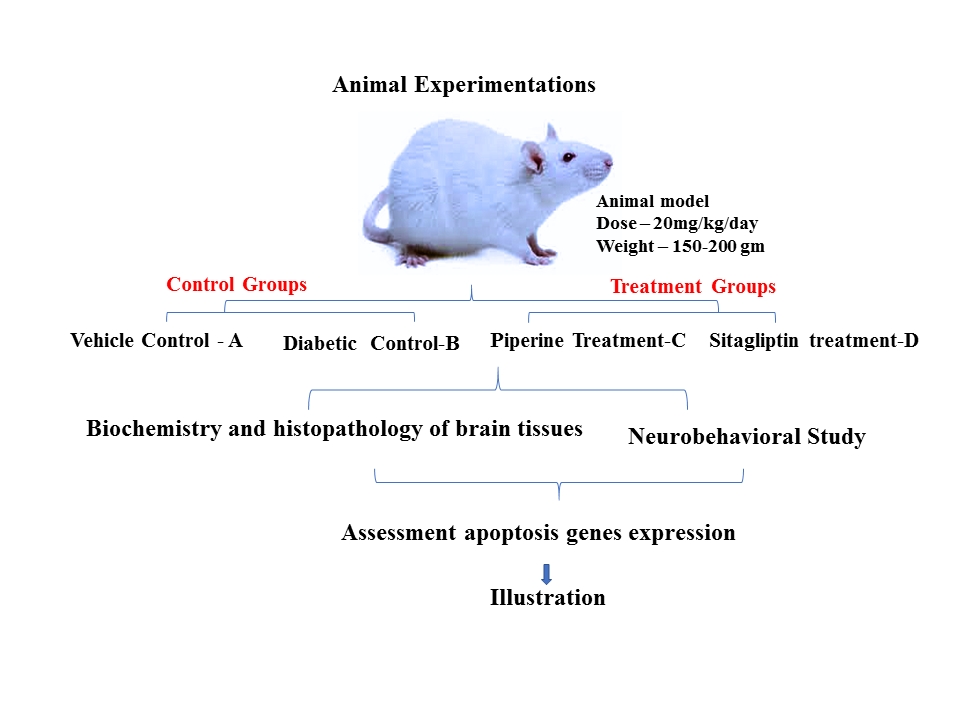

Supplement: Supplementary file 1 [file image1.jpeg]
